# Pro-atherosclerotic disturbed flow disrupts caveolin-1 expression, localization, and function via glycocalyx degradation

**DOI:** 10.1186/s12967-018-1721-2

**Published:** 2018-12-18

**Authors:** Ian C. Harding, Ronodeep Mitra, Solomon A. Mensah, Ira M. Herman, Eno E. Ebong

**Affiliations:** 1 Department of Bioengineering, Northeastern University, Boston, MA USA; 2 Department of Chemical Engineering, Northeastern University, Boston, MA 02115 USA; 3 Department of Developmental, Molecular, and Chemical Biology, Tufts Sackler School of Graduate Biomedical Sciences, Boston, MA USA; 4 Center for Innovations in Wound Healing Research, Tufts University School of Medicine, Boston, MA USA; 5 Department of Neuroscience, Albert Einstein College of Medicine, New York, NY USA

**Keywords:** Atherosclerosis, Endothelial cells, Fluid shear stress, Glycocalyx, Caveolin, Endothelial-type nitric oxide synthase

## Abstract

**Background:**

Endothelial-dependent atherosclerosis develops in a non-random pattern in regions of vessel bending and bifurcations, where blood flow exhibits disturbed flow (DF) patterns. In contrast, uniform flow (UF), normal endothelium, and healthy vessel walls co-exist within straight vessels. In clarifying how flow protectively or atherogenically regulates endothelial cell behavior, involvement of the endothelial surface glycocalyx has been suggested due to reduced expression in regions of atherosclerosis development. Here, we hypothesized that pro-atherosclerotic endothelial dysfunction occurs as a result of DF-induced reduction in glycocalyx expression and subsequently impairs endothelial sensitivity to flow. Specifically, we propose that glycocalyx degradation can induce pro-atherosclerotic endothelial dysfunction through decreased caveolin-1 and endothelial nitric oxide synthase expression and localization.

**Methods:**

We studied endothelial cells in atherosclerotic-prone DF and atherosclerotic-resistant UF conditions in parallel plate flow culture and in C57Bl/6 mice. The effects of flow conditioning on endothelial cell behavior were quantified using immunocytochemistry. The glycocalyx was fluorescently labeled for wheat germ agglutinin, which serves as a general glycocalyx label, and heparan sulfate, a major glycocalyx component. Additionally, mechanosensitivity was assessed by immunocytochemical fluorescence expression and function of caveolin-1, the protein that forms the mechanosignaling caveolar invaginations on the endothelial surface, total endothelial-type nitric oxide synthase (eNOS), which synthesizes nitric oxide, and serine 1177 phosphorylated eNOS (eNOS-pS1177), which is the active form of eNOS. Caveolin function and eNOS expression and activation were correlated to glycocalyx expression. Heparinase III enzyme was used to degrade a major glycocalyx component, HS, to identify the role of the glycocalyx in caveoin-1 and eNOS-pS1177 regulation.

**Results:**

Results confirmed that DF reduces caveolin-1 expression and abolishes most of its subcellular localization preferences, when compared to the effect of UF. DF down-regulates caveolin-1 mechanosignaling, as indicated by its reduced colocalization with serine 1177 phosphorylated endothelial-type nitric oxide synthase (eNOS-pS1177), a vasoregulatory signaling molecule whose activity is regulated by its residence in caveolae. As expected, DF inhibited glycocalyx expression compared to UF. In the absence of heparan sulfate, a major glycocalyx component, UF-conditioned endothelial cells exhibited near DF-like caveolin-1 expression, localization, and colocalization with eNOS-pS1177.

**Conclusions:**

This is the first demonstration of a flow-defined role of the glycocalyx in caveolae expression and function related to vasculoprotective endothelial mechanosensitivity that defends against atherosclerosis. The results suggest that a glycocalyx-based therapeutic targeted to areas of atherosclerosis development could prevent disease initiation and progression.

**Electronic supplementary material:**

The online version of this article (10.1186/s12967-018-1721-2) contains supplementary material, which is available to authorized users.

## Background

The burden of atherosclerosis, which contributes to strokes, heart attacks, and other cardiovascular diseases, remains tremendous despite significant advances in disease prevention and treatment [1]. Despite an improved knowledge of atherosclerosis development and therapeutics capable of combatting the disease, mainly statins, the prevalence of atherosclerosis-derived events remains high. Therefore, improved therapeutics that more efficiently and widely prevent atherosclerosis development are desired.

Atherosclerosis disease mechanisms are multifactorial. Blood vessel geometry is one important factor, as indicated by the development of atherosclerosis in a vascular site-specific manner [2, 3]. Most often, atherosclerotic plaques appear in vessel regions of high curvature or branches, such as the carotid bifurcation and aortic arch [2–5]. The complex geometry in these regions is known to create disturbed flow (DF) profiles [2–5]. In the carotid bifurcation the profile is characterized by low time-averaged shear stress magnitudes, high shear stress gradients, and occasional flow reversal [2–5]. Atherosclerotic plaques almost never localize in long, straight arteries, such as the abdominal aorta, which exhibit uniform flow (UF) that is unidirectional with constant and relatively high time-averaged shear stress magnitudes [2, 3]. These observations, taken together, are strong indications of the relationship between local hemodynamics and atherosclerotic lesion progression [5–7].

Another widely recognized factor that contributes to atherosclerosis is pathological change in the vascular endothelium, which precedes disease development [8–10]. Alterations in endothelium function, which can actually vary quite distinctly from vasculature of different origins [11], include deactivation of anti-atherogenic signaling pathways and activation of pro-atherogenic signaling, impaired vasoregulation, transition from an anti- to a pro-inflammatory state, which includes increased expression of adhesion molecules, and elevated permissiveness to small molecules and whole cells crossing the vascular wall [12–14]. Studies conducted over the last three or more decades have confirmed hemodynamic force, most interestingly shear stress, as an important regulator of endothelium behavior [15]. Both in vitro and in vivo studies have demonstrated that UF characteristic of atheroprotective regions of the vasculature promotes endothelial homeostasis, while atheropermissive DF induces endothelial dysfunction.

Mechanosensitivity of the vascular endothelium, both of macro- and microvasculature [16], and subsequent changes in phenotype have been attributed to a number of mechanotransduction pathways. Caveolae, for example, are well-known mechanosignaling invaginations of the cell membrane structures, that are rich in cholesterol, sphingolipids, and a variety of signaling molecules [17–19]. To our knowledge, there is no prior information on atheropermissive DF regulation of caveolae and caveolin-1 (cav-1), the protein that coats caveolae. However, atheroprotective UF, in comparison to static flow conditions, has been shown to increase expression of caveolae and cav-1, while also increasing caveolae/cav-1 polarization to the portion of endothelial cells (ECs) that lies upstream of the flow direction [20, 21]. In healthy conditions, caveolae formation at the EC membrane, caveolae ability to receive extracellular stimuli and induce intracellular responses, and, subsequently, diverse caveolae-derived signaling cascades, make the membrane invaginations essential signaling domains that assist in maintaining endothelial homeostasis. Improper expression of caveolae can therefore lead to endothelial dysfunction relevant to atherosclerosis development.

For example, in one study, loss of caveolae in genetically modified mice was found to impair endothelium-dependent regulation of vascular relaxation, contractility, and myogenic tone [22]. Other studies have implicated caveolae in regulating the expression of the anti-atherogenic vascular relaxation factor, nitric oxide (NO), which is enzymatically produced by endothelial-type NO synthase (eNOS) [23]. Lipid modification of eNOS, by myristoylation and palmitoylation, can target the enzyme to caveolae [19, 24] where it is activated through Ca^2+^ exposure or through Ca^2+^ independent phosphorylation at Serine 1177 [25–27]. Activated eNOS is then enabled to produce and release NO, upon uncoupling from the cav-1 scaffold protein [28–31]. Prior studies have collectively demonstrated that UF conditions can sustain this anti-atherogenic, caveolae-derived signaling cascade [32–35]. While eNOS and NO expression are known to be suppressed in atheroprone regions of the vasculature [33–36], the direct connection between DF and the signaling cascade that starts with caveolae and cav-1 expression and ends with eNOS activation and NO release remains to be clarified.

Protective or atherogenic fluid mechanotransduction regulation of caveolae/cav-1 expression and mechanosignaling function may involve the EC surface glycocalyx (GCX) [37]. GCX is a polysaccharide mesh layer on the outer surface of the endothelial membrane. The GCX consists of sialic acid and glycosaminoglycans, such as heparan sulfate (HS), hyaluronic acid, and chondroitin sulfate, some of which bind to the syndecan and glypican membrane-attached proteoglycan core proteins [38, 39]. HS is the most abundant GCX glycosaminoglycan and, for the present study, HS is of particular interest because it binds to the glypican-1 core protein that, along with eNOS, resides in mechanosignaling caveolae [40, 41]. Due to its unique location at the interface between the EC surface and the extracellular environment, GCX has been implicated in sensing and converting physiological fluid shear stress and mechanical stretch into biochemical signals to support healthy endothelial phenotypes [41–43]. Most interestingly, GCX degradation has been linked to endothelial-dependent atherosclerosis and a number of other diseases while a healthy GCX has been shown to promote proper endothelium function [44–47]. For example, evidence from in vivo and in vitro studies confirm that UF-conditioned GCX is abundant [37, 48–50] and its HS component, via increased glypican-1 in caveolae, is responsible for eNOS activation and flow-induced NO production [51–56]. In contrast, atheroprone DF conditions have been shown to be less supportive of GCX expression [37, 48–50]. This decreased GCX expression has also been associated with endothelial motility and proliferation [57], increased vascular permeability [58], and leukocyte adhesion [59]. However, downstream in the mechanotransduction pathway, GCX-mediated DF regulation of caveolae and its eNOS/NO relevant mechanosignaling function has never been studied, to our knowledge.

The goal of the present study is to link well-defined physiologically accurate flow conditions to GCX-mediated flow-regulation of caveolae/cav-1 and activated eNOS, which are relevant to atherosclerosis prevention or onset. We hypothesized that DF patterns will lead to decreased cav-l expression, loss of cav-1 cellular compartment-specific localization, and decreased cav-1 colocalization with activated eNOS, all due to suppressed GCX. Our results may reveal a mechanism by which GCX integrity contributes to atherosclerosis prevention while GCX damage permits disease progression, suggesting that the GCX may be a viable target for next-generation atherosclerosis prevention and treatment approaches.

## Materials and methods

All materials were purchased from Fisher Scientific, MA, USA unless otherwise noted.

### Cell culture

Rat fat pad ECs (RFPECs) were previously isolated from rat epididymal fat pad, immortalized, and shared with us by Dr. Mia Thi of the Albert Einstein College of Medicine (Bronx, NY, USA). RFPECs were cultured at passages 20 to 60 in Dulbecco’s Modified Eagle Medium (DMEM) with 10% fetal bovine serum (FBS) and 1% penicillin–streptomycin (P/S). Cells were grown at 37 °C in a humidified incubator maintained with 5% CO_2_. For shear stress experiments (described below), cells were plated on fibronectin-coated (3 μg/mL) glass coverslips (#1.5 or 0.17 mm thickness) at a seeding density of approximately 3 × 10^3^ cells/cm^2^. Cells were cultured in static conditions for 2 to 4 days until fully confluent. At confluence, cells were transferred to flow conditions in GCX-stabilizing medium containing reduced FBS (5%) along with bovine serum albumin (BSA; 0.5%).

It is important to note here that RFPECs are ideal for this study, because unlike many other EC types RFPECs express a robust GCX [60, 61]. Furthermore, similar to other EC types, RFPECs properly respond to the application of shear stress [62, 63] and have been shown to elicit atherosclerosis relevant GCX function [64–67], F-actin expression, morphology, and alignment [62, 68], intercellular communication [62, 69], endothelium permeability [70, 71], and NO production [72]. Acknowledging that ECs are heterogenous due to their origins from different species and different vascular beds [73], we wish to indicate that we are conducting parallel studies with primary endothelium originating from other species, including human, and from a variety of vascular beds, including the aorta and coronary arteries. The additional investigations, taken together with this one, will provide comprehensive information, are far beyond the scope of this report, and will be described in other companion publications.

### Shear stress apparatus

The confluent RFPECs were exposed to shear stress in a custom, parallel-plate flow chamber that was designed using SolidWorks and SolidWorks Flow Simulation. The chamber was designed to simulate atherosclerotic-prone and atherosclerotic-resistant flow patterns in adjacent neighboring spatial regions of the carotid artery (Additional file 1: Fig. S1a). Briefly, the device was an enclosed rectangular chamber with a height that was much smaller than the width and length. The chamber consisted of a top, a bottom, a rubber gasket to form a seal and shape the flow channel, and the coverslip coated with ECs on fibronectin (Additional file 1: Fig. S1b). To assemble the apparatus, the coverslip was placed on the chamber bottom, and the chamber top was then screwed to the chamber bottom with the rubber gasket in between. The flow apparatus was then connected to a peristaltic pump. Cells were exposed to 6-h of flowing DMEM containing 5% FBS, 1% P/S, and 0.5% BSA. Generally, the flow pattern was uniform (UF), as associated with atherosclerosis resistance. However, for our study, UF was interrupted by a protuberance at the bottom of the chamber to produce pro-atherosclerotic DF. This flow is characterized by separation followed by recirculation, stagnation, and eventual recovery to UF. During flow perfusion, the shear stress apparatus was maintained in an incubator at 37 °C with 5% CO_2_ and humidity, an environment that the flow apparatus can withstand due to its fabrication out of 316 L stainless steel. The stainless steel is also biocompatible, which protects the ECs and DMEM from toxicity. An open media reservoir was placed upstream of the pump, to ensure proper CO_2_ diffusion into the flow system’s DMEM. We also inserted a pulse dampener directly after the pump and upstream of the chamber inlet, to eliminate pulsatility generated by the peristaltic pump. Further details about the shear stress apparatus design and validation are elaborated upon later in this report.

### Glycocalyx treatment

The RFPEC GCX was generally treated with the aforementioned flow conditions—DF versus UF—as generated by the flow apparatus. The RFPEC GCX was also treated by static flow, which served as a control condition. To confirm the role of the GCX in any observed RFPEC response to flow, in some experiments the GCX was also chemically treated with the enzyme heparinase III (Hep III; Ibex, Quebec, Canada). Hep III exclusively cleaves HS glycosaminoglycans, a major GCX component, thus impairing the GCX layer. RFPECs in both static and UF conditions were exposed to 62.5 micro-international units per milliliter (µIU/mL) of Hep III in DMEM for 6 h, allowing for simultaneous flow application and enzyme treatment. The Hep III concentration was determined via dose–response pilot experiments to degrade RFPEC HS by ~ 50% (data shown in Additional file 2: Fig. S2). For further clarification of when and why flow and Hep III were used to treat the GCX, refer to Table 1.

**Table 1:** GCX flow and/or chemical treatment with rationale, where applicable

		Chemical stimulus		Rationale
		Untreated	Hep III-treated	
Flow stimulus	Static	Yes	Yes	To assess whether GCX controls baseline EC behavior (as opposed to flow-induced EC behavior)
	Uniform	Yes	Yes	To assess whether EC behavior in UF only occurs due to endothelium coverage by the GCX
	Disturbed	Yes		
Rationale		To assess GCX expression and EC behavior in response to both pro- and anti-atherosclerotic flow patterns		

### Animal studies

All animal studies were carried out using procedures approved by the Northeastern University Institutional Animal Care and Use Committee (IACUC). Male C57Bl/6 mice were obtained from Jackson Laboratories, fed a chow diet, and studied at 6–8 weeks of age.

In one experiment, acute DF was induced in vivo, by surgically performing a partial ligation of the left carotid artery (LCA), as previously described [74]. This allowed for the investigation of DF effects on EC behavior in vivo. Briefly, mice were anesthetized with isoflurane and their LCAs were exposed through a 4–5 mm ventral midline incision followed by blunt dissection. Three of the four caudal branches of the LCA, including the left external carotid, internal carotid, and occipital artery, were ligated with 6–0 silk sutures, leaving the fourth branch (superior thyroid artery) intact (Fig. 4a). The incision was then closed. The mice were briefly monitored to confirm a successful recovery, and kept alive for a week to allow in vivo DF to induce endothelial dysfunction [74]. To investigate UF effects on EC behavior in vivo, surgery was not performed and LCA was left non-ligated. One week after surgery, ligated and non-ligated LCAs were isolated from mice that were initially anesthetized using isoflurane and followed by xylazine (20 mg/mL) and ketamine (100 mg/mL). Mice vessels were then drained of blood by perfusion with a 1% BSA solution in phosphate buffered saline (PBS), administered via the left ventricle after severing the inferior vena cava. Following this, mice were perfusion fixed with a 2% paraformaldehyde solution in PBS. The ligated and non-ligated LCAs were isolated after removal of periadventitial fat. LCAs were then embedded in Tissue-Tek optimum cutting temperature (OCT) medium, frozen at − 80 °C, and 6-µm axial sections were made using a Leica CM3050 cryostat.

In another experiment, effects of chronic in vivo DF were assessed in aortas of chow diet fed 6–8-week-old male C57Bl/6 mice. As described by previous reports, ECs along the aortic arch and descending aorta, specifically along the inner curvature, are exposed to low shear stresses and DF that can predispose the vessel to atherosclerosis [75, 76]. In contrast, the proximal abdominal aorta experiences fully developed UF, leading to atherosclerosis resistance. To extract mouse aortas, mice were processed as described above. Aortas were cleared of perivascular fat, dissected, kept in PBS at 4 °C for a maximum of 2 days, fluorescently labeled as described below, and longitudinally cut using curved 8-cm vannas scissors and a dissection microscope for *en face* imaging [77].

### Fluorescent staining of caveolin-1, eNOS, and glycocalyx in vitro

For in vitro GCX staining immediately after exposure to static or flow conditions, RFPECs with untreated or Hep III-treated GCX were fixed using 2% paraformaldehyde and 0.1% glutaraldehyde in PBS for 30 min (PBS). For a general label of the GCX, RFPECs were stained using wheat germ agglutinin (WGA), which targets GCX constituents that include sialic acid and *N*-acetylglucosamine, a component of HS and hyaluronic acid [38, 78]. To accomplish WGA staining, RFPEC monolayers were blocked with 3% BSA for 30 min at room temperature and then incubated in biotinylated WGA (1:100, Vector Labs, CA, USA) for 1 h at 4 °C. For secondary labeling, Alexa Fluor 488-conjugated streptavidin was used at a concentration of 1:1000 for 30 min at 4 °C. To specifically label the HS component of the GCX, RFPECs were blocked in 2% goat serum for 30 min at room temperature and incubated in mouse anti-HS (1:100, 10E4 epitope, Amsbio, MA, USA) overnight at 4 °C. Secondary labeling was achieved by incubating cells with goat anti-mouse conjugated with Alexa Fluor 488 (1:1000) for 30 min at room temperature.

For in vitro cav-1 and eNOS staining, RFPEC monolayers were fixed with 4% paraformaldehyde in PBS for 20 min. RFPECs were then permeabilized using a 0.2% Triton X-100 solution for 5 min and blocked in 10% goat serum for 30 min at room temperature. RFPECs were co-incubated with rabbit anti-eNOS specific to eNOS phosphorylated on the 1177 serine residue (eNOS-pS1177) (1:500, Signalway Antibody, MD) and mouse anti-cav-1 (1:200, Santa Cruz Biotechnology, TX) overnight at 4 °C. This was followed by secondary incubation with goat anti-rabbit labelled with Alexa Fluor 647 (1:200, cav-1) and goat anti-mouse tagged with Alexa Fluor 488 (1:200, eNOS-pS1177), respectively, for 1 h at room temperature.

All fixed and stained RFPECs were then coverslipped using VectaShield anti-fade mounting medium with 4′,6-diamidino-2-phenylindole (DAPI) for nuclear staining (VWR, PA, USA).

### Fluorescent staining of caveolin-1, eNOS, and glycocalyx in vivo

For in vivo staining of 6-µm cryosectioned LCAs, samples were post-fixed for 10 min with 4% PFA and permeabilized with 0.3% Triton X-100 for 10 min. Antigen retrieval was subsequently performed in 1.2 mM sodium citrate by pressure cooking for 10 min. To block endogenous peroxidase for future tyramide signal amplification, samples were incubated with 1% H_2_O_2_ for 10 min. Samples were then blocked using 10% goat serum for 1 h and incubated with either biotinylated WGA or rabbit anti-cav-1 (1:50) overnight at 4 °C. For fluorescent detection, samples were then labeled with horseradish peroxidase (HRP)-conjugated streptavidin or HRP-conjugated goat anti-rabbit (1:500) for 1 h at 4 °C. Tyramide signal amplification using the cyanine 3 (Cy3) fluorophore was then performed using a PerkinElmer Cy3 TSA kit following the manufacturers protocol. Samples were then mounted in VectaShield mounting medium with DAPI.

Mouse aortas were labeled for WGA, cav-1, and total eNOS using a protocol similar to the in vitro fluorescent staining procedure. For WGA, in 24-well plates, 3–4 mm aortic segments were blocked with 3% BSA, incubated in biotinylated WGA for 1 h at 4 °C, and then incubated in Alexa Fluor 488-conjugated streptavidin for 30 min at room temperature. For cav-1 and total eNOS, samples were blocked with 5% goat serum for 1 h at room temperature, separately incubated with either rabbit anti-cav-1 (1:300, Thermo Fisher Scientific, MA) or rabbit anti-total eNOS (1:200, Cell Signaling Technology, MA) overnight at 4 °C, and then incubated with Alexa Fluor 488 conjugated goat anti-rabbit secondary at 1:500 (cav-1) or 1:1000 (total eNOS) for 1 h at room temperature. All steps at room temperature were performed on a rocker. Aortic segments were placed in VectaShield mounting medium containing DAPI and then longitudinally cut using a dissection microscope and mounted on glass coverslips as previously described [77].

### Confocal microscopy

All RFPECs, LCA cryosections, and *en face* aorta vessel sections were imaged using a Zeiss LSM 710 laser scanning confocal microscope at 40× or 63× magnification (with an oil immersion lens) with 8-bit pixel values for each color channel. Gain and laser power levels were set for each experiment to provide adequate signal levels below saturation point. Microscope settings remained the same throughout data collection for each experiment. Images of XY-plane slices were collected and then stacked to create a Z-projection image.

### Data acquisition

All data acquisition was performed using ImageJ, unless otherwise specified.

First, to determine overall expression levels of cav-1 and eNOS in RFPECs, ImageJ was used to calculate the mean fluorescence intensity (MFI) of the respective immunofluorescence signals from *en face* images of the Z-projections. Over a specified field of view, MFI provides a measure of the average pixel value, ranging from 0 to 255, for each color channel. In all cases, a field of view was taken as an entire 63× magnification *en face* image.

It was previously noted that cav-1 localization correlates to direction of pressure drop and corresponding flow [20]. Therefore, cav-1 distribution with respect to flow direction was examined, as previously described [20]. In the studies performed in vitro, randomly selected RFPECs were identified to have preferential cav-1 localization in one of four zones of relatively equal size. These zones were defined as upstream (towards the direction of flow), downstream (away from the direction of flow), upper lateral region, and lower lateral zones. An additional, fifth “zone” was also considered, in which cav-1 was evenly dispersed.

To quantify cav-1 localization at cell membranes, where the protein can contribute to the formation of caveolae rafts, kurtosis analyses were performed in the in vitro studies of RFPECs. The kurtosis analysis provides a coefficient indicative of the degree to which immunofluorescence data is centralized to a specific point. Therefore, performing kurtosis analyses between two neighboring ECs provided us with a measure of the density of cav-1 immunofluorescence at the cell-to-cell appositions in comparison to the intracellular compartments of ECs. Kurtosis coefficient values for various experimental conditions were normalized to the kurtosis values of the static control condition. Normalized kurtosis values below 1.0 reflected decentralization of cav-1 from the cell membranes, while values above 1.0 reflected increased cav-1 centralization to cell membranes.

Colocalization of caveolae-associated signaling molecules, represented by activated eNOS, to caveolae was assessed after eliminating background noise in Z-projection *en face* images of RFPECs. To eliminate background noise, a signal intensity threshold was determined by measuring fluorescence intensity within the field of view at positions above the cell surface. Pixels with signal intensities at or below the threshold were removed. With background noise subtracted, colocalization was determined using the Colocalization Threshold tool on ImageJ. The colocalization values were then analyzed using the Mander’s overlap coefficient approach to quantify the extent to which activated eNOS overlaps cav-1. Mander’s method was employed in lieu of other colocalization analysis techniques, such as the Pearson’s method, because of the localization of eNOS in multiple areas of the cell and the large variation in the ratio of fluorescent probe signal intensity [79].

It was of interest to determine if GCX correlated to and was involved in cav-1 function. Therefore, overall levels of GCX expression in RFPECs were determined using ImageJ by calculating MFI of GCX immunofluorescence in the same manner that was used for determining eNOS and cav-1 expression. In addition to examining GCX expression, GCX thickness was also assessed. Measurements of GCX thickness were performed from cross-sectional views of the confocal z-projection images. On the cross-section view, randomized areas of the cell membrane were chosen using the “Grid” tool. Lines at the predetermined randomized positions were then drawn perpendicular to the apical cell membrane, which contained immunofluorescence signal representing the GCX (Additional file 3: Fig. S3). The fluorescent profile along the line was then plotted (Additional file 3: Fig. S3). On the fluorescence profile plots, the width of fluorescence signal above background signal was identified and taken to indicate GCX thicknesses (Additional file 3: Fig. S3).

Confocal microscope fluorescence images of LCAs and aortas stained for cav-1, total eNOS, and WGA-labeled GCX were qualitatively assessed for the most part. Quantitative analysis of LCA and aorta images, although limited, were performed in accordance with RFPEC analysis as described above.

### Statistical analysis

Prior to statistical analyses, ImageJ data points were normalized with respect to static samples within each experiment. Normalization within experiments eliminated any bias caused by microscopic and other variations between experiments. Therefore, experimental data acquired from ImageJ quantification are presented in the “Results” section as normalized means ± standard error of the means (SEMs). The total number of experiments, along with counts of the RFPEC monolayer replicates and sizes of data sets within each experiment, are also presented in the “Results” section. These experimental data were statistically analyzed using GraphPad Prism software. Upon confirming uniform distributions of the data sets collected from static flow, DF, UF, static flow with Hep III treatment, or UF with Hep III treatment conditions, one-way ANOVA analyses were performed. In addition, post hoc Tukey’s multiple comparison tests were completed with an alpha value of 0.05, unless otherwise specified.

## Results

### Generation and characterization of atherosclerosis-relevant flow in vitro

As designed using SolidWorks and SolidWorks Flow Simulation, our custom parallel-plate flow chamber produced a physiologically relevant flow pattern, which specifically mimicked the flow patterns experienced at the carotid artery bifurcation, a common area of atherosclerosis development (Additional file 1: Fig. S1d) [4, 5, 80]. After a number of SolidWorks iterations to characterize the flow profiles of various channel- and protuberance-relative dimensions, the final chamber contained a 90 mm long, 13.5 mm wide, and 1 mm high flow channel (Additional file 1: Fig. S1d). To generate a pro-atherosclerotic, DF waveform, a 0.35 mm high protuberance was inserted within this channel directly upstream of the cell monolayer (Additional file 1: Fig. S1d). The SolidWorks Flow Simulations were run using advanced narrow channel mesh refinement, by modeling all surfaces as no-slip flow boundaries, and with an inlet volumetric flow rate set at 180 mL/min and outlet pressure set at 1 atmosphere. Based on the finalized channel with the aforementioned protuberance dimensions, the SolidWorks Flow Simulation model revealed the creation of DF patterns directly downstream of the protuberance, spanning just over 1.0 cm in length (Additional file 1: Fig. S1c). After flow separation, along the bottom surface of the flow channel, the DF region was shown to contain the following areas: flow recirculation with non-uniform magnitudes of shear stress ranging from − 8 dynes/cm^2^ to 0 dynes/cm^2^ located at 0 to 0.2 cm downstream of the step, flow reattachment with 0 dynes/cm^2^ shear stress and located at 0.2 cm, and flow recovery ranging between 0 and 12 dynes/cm^2^ shear stress in the 0.2 to 1.0 cm area (Additional file 1: Fig. S1c). A fully developed flow region, located beyond 1.0 cm downstream of the step, was observed to have a uniform shear stress value of approximately 12 dynes/cm^2^ (Additional file 1: Fig. S1c).

Based on the final flow chamber design and flow pattern, it was determined that DF conditioned cells should be imaged and experimentally examined near the flow reattachment region at approximately 0.18 cm downstream of the step, corresponding with a shear stress of ~ 1 dynes/cm^2^. UF conditioned cells were examined at 2.0 cm or farther downstream of the step, at 12 dynes/cm^2^.

For further details about the flow generation and characterization results, refer to our previous publication [81], which reports on the design of a similar flow apparatus.

### Flow-induced caveolin-1 expression is attenuated in disturbed flow in vitro and in vivo

**Fig. 1:**
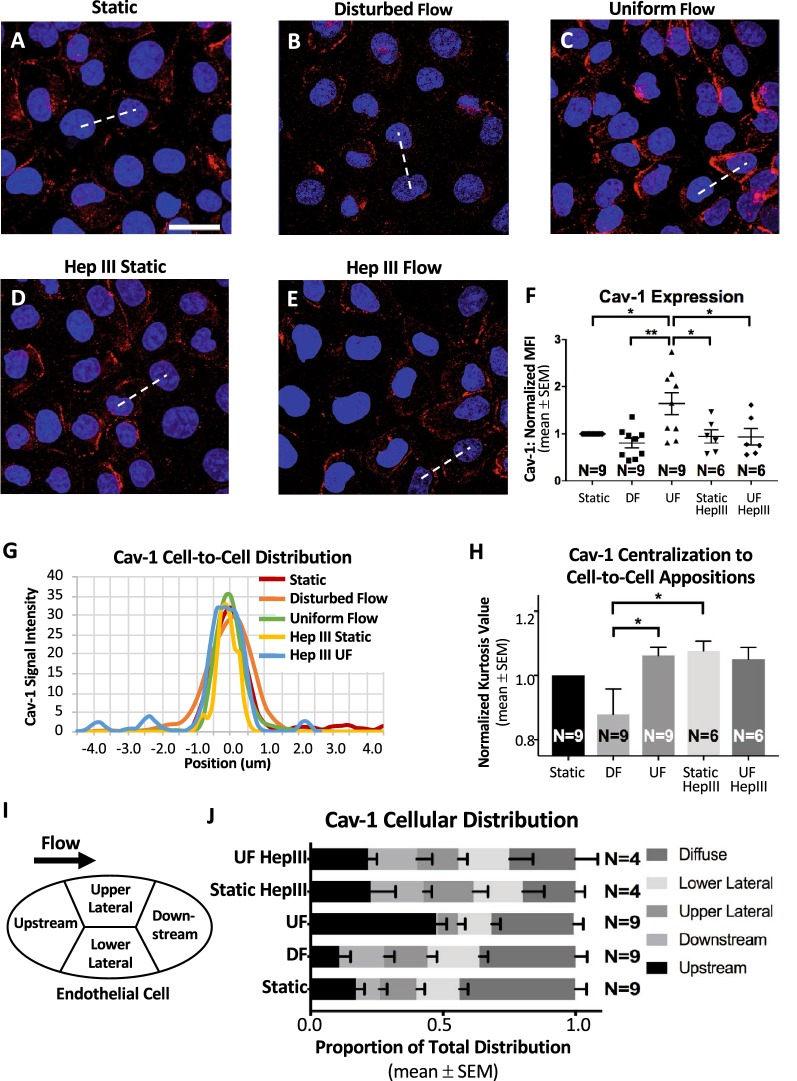
Heparan sulfate regulates flow-induced cav-1 expression and, partially, cav-1 localization. Cav-1 immunofluorescence of RFPECs is shown for conditions of **a** static flow, **b** DF, **c** UF, **d** static flow with Hep III treatment, and **e** UF with Hep III treatment (scale bar: 30 μm). **f** Quantification of cav-1 MFI demonstrates a significant highest expression of cav-1 in UF conditioned cells compared to all other conditions. **g** Representative cav-1 signal intensity curves showing, for each experimental condition, the distribution of cav-1 from one cell to a neighboring cell. The signal intensity curves were determined along equidistant lines (see representative hatched lines) that connect the intracellular compartments of the neighboring cells and the cell-to-cell appositions are at the 0.0 μm positions along each line. On first glance, distribution of cav-1 looks consistent for all conditions (**h**) Kurtosis analysis of full sets of these curves clarifies that indeed there are small differences in cav-1 distribution. However, it is important to note that RFPECs exposed to UF exhibit statistically more appositional localization of cav-1 than RFPECs exposed to DF, and there is no strong statistical evidence that HepIII treatment impacts this difference in cav-1 distribution. **i** Schematic view of the subcellular zones that were examined to assess preferential cav-1 localization. To clarify, preferential localization of cav-1 was determined based on MFI and not kurtosis analysis. **j** Compared to all other experimental conditions, exposure to UF induces significantly higher localization of cav-1 in the subcellular region that lies upstream with respect to flow direction. **f**, **h**, **j** Number of independent experiments “N” are shown for each condition, and *P < 0.05, **P < 0.01, ***P < 0.001, and ****P < 0.0001 are indicated in this Fig. 2 or in the accompanying Additional file 4: Fig. S4

To determine the effects of DF on endothelial function, we first investigated in vitro flow-dependent expression of the mechanosignaling caveolar invaginations on the EC surface. To do so, we specifically assessed the caveolae coating protein cav-1, because previous studies correlated caveolae presence to the expression of cav-1 and other caveolin coat proteins [22, 82–84]. In static conditions RFPECs displayed an average cav-1 MFI of 20.3 ± 3.34 relative fluorescent units (RFUs), normalized to a value of 1 (Fig. 1a, f). Comparatively, UF caused a statistically significant 63.9 ± 23.2% increase in cav-1 MFI, reaching 28.9 ± 4.35 RFUs. DF, with an average cav-1 MFI of 15.7 ± 3.51 RFUs, showed a statistically insignificant 19.3 ± 10.5% decrease in cav-1 MFI (Fig. 1b, f) compared to static conditions. Furthermore, when compared to UF, cells exposed to DF expressed 49.6 ± 10.5% less cav-1, which was statistically significant (Fig. 1b, c, f).

To confirm these in vitro findings of flow-dependent cav-1 expression in vivo, partially ligated mouse LCAs experiencing acute DF conditions that induce accelerated pre-atherosclerotic endothelial dysfunction were compared to non-ligated LCAs that largely experience UF (Fig. 4a) [74]. Qualitative assessment revealed that cav-1 in non-ligated LCAs that experience UF is upregulated, compared to ligated LCAs that experience DF (Fig. 4b, c). This is similar to what we found in vitro for RFPECs. When quantitatively analyzed and compared to DF, we observed a 55.1 ± 12.0% increase in cav-1 expression in non-ligated LCAs exposed to UF.

We also investigated the expression of cav-1 in the aortic arch mouse vessel, where chronic pro-atherosclerotic DF conditions exist, and we compared it to cav-1 expression in the abdominal aorta vessel region, where UF conditions are present (Fig. 4f) [75, 76]. Again, we found more cav-1 in UF than in DF (Fig. 4g, h). Specifically, cav-1 in vivo was increased by 1.78-fold in the abdominal aorta when compared to the aortic arch. Collectively, these preliminary in vivo results reiterate our in vitro results and support the fact that regulation of cav-1 is flow-dependent.

### Caveolin-1 distribution preferences are flow-dependent

In addition to the flow associated changes in overall cav-1 expression, we observed in vitro that there are flow-dependent differences in the cellular distribution of cav-1. Cellular distribution was determined in RFPECs using previously specified zones, which were defined as upstream of the direction of flow, downstream of the direction of flow, upper lateral, lower lateral, or dispersed (Fig. 1i). A statistically significant majority of RFPECs in static conditions, 43.7 ± 4.32%, contained diffusely expressed cav-1 (Fig. 1j). In cells exposed to UF, as expected [20], a statistically significant 47.4 ± 3.92% majority of cells preferentially expressed cav-1 at the upstream zone (Fig. 1j). This corresponds to a 178 ± 3.92% increase in upstream localization compared to static samples. On the other hand, cells exposed to DF showed no preferential localization of cav-1 to a specific cellular zone. Instead, DF cells expressed diffuse cav-1 34.8 ± 4.55% of the time, which was statistically similar to static controls and statistically different from UF conditions (Fig. 1j).

Preferential localization of cav-1 to the RFPEC borders where it can form a caveolae coat also depended on flow conditions. The normalized kurtosis value for static control ECs, which represents the degree of cav-1 localization at EC-to-EC appositions, was compared to the normalized kurtosis value for ECs in UF. This comparison revealed 6.20 ± 2.63% more localization of cav-1 to the appositions between UF-treated cells than to the appositions between static-treated cells (Fig. 1g, h). In another comparison, 12.01 ± 7.88% less cav-1 was distributed to cell appositions in DF-treated cells compared to static conditions (Fig. 1g, h). These differential flow effects, when compared, showed that DF-conditioned cells exhibited 17.1 ± 7.88% less localization of cav-1 to cell borders than UF-conditioned cells, which was statistically significant (Fig. 1g, h).

### Flow-induced caveolin-1 colocalization with the eNOS signaling molecule is decreased in disturbed flow in vitro and in vivo

As previously mentioned, signaling molecules such as eNOS are ready for activation when taken up and stored by caveolae [31, 85]. This knowledge motivated the goal of assessing potential caveolae function by quantifying cav-1 colocalization with a representative signaling molecule, eNOS, which produces NO when activated. To fulfill this goal, in RFPECs we assessed expression of active eNOS and analyzed eNOS overlap with cav-1.

**Fig. 2:**
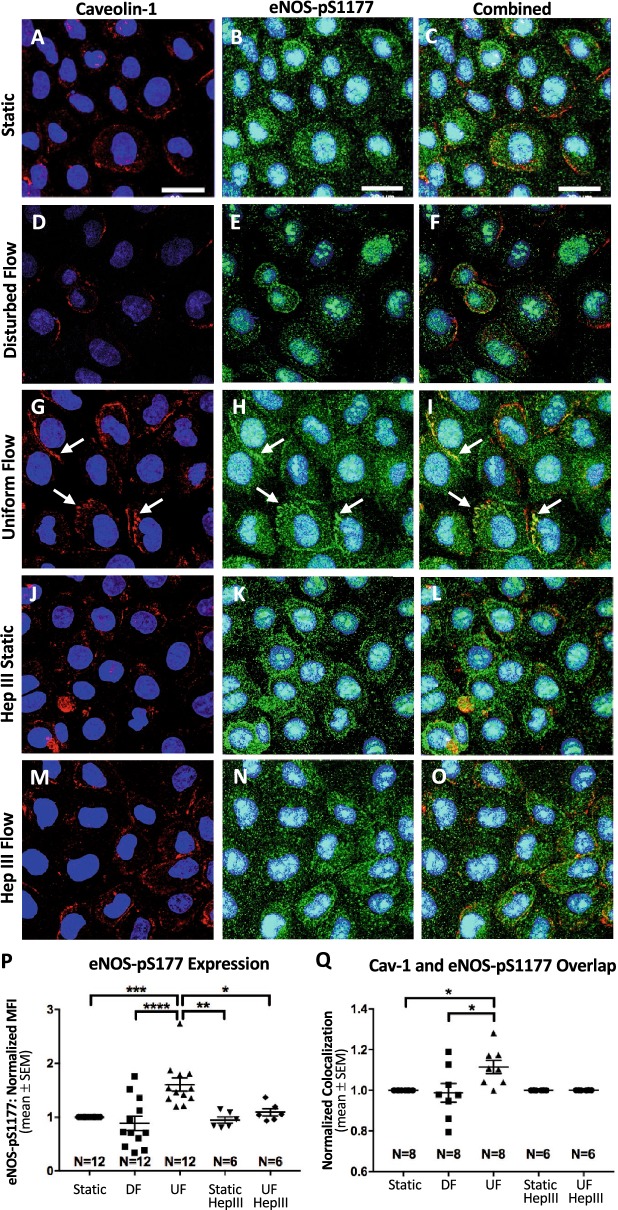
Heparan sulfate regulates flow-mediated RFPEC eNOS-pS1177 expression and colocalization with cav-1. En face z-projections are shown for red fluorescent cav-1 (**a**, **d**, **g**, **j**, **m**), green fluorescent eNOS-pS1177 (**b**, **e**, **h**, **k**, **n**), and merged cav-1 with eNOS-pS1177 (**c**, **f**, **i**, **l**, **o**). Conditions examined include static flow (**a**–**c**), DF (**d**–**f**), UF (**g**–**i**), static flow with Hep III treatment (**j**–**l**), and UF with Hep III treatment (**m**–**o**). Arrows are pointing to areas of significant cav-1 colocalization with eNOS-pS1177. Scale bar = 30 μm. **p** Quantification of eNOS-pS1177 expression. Compared to static controls, cells with intact HS display decreased eNOS-pS1177 expression when exposed to DF, but increased eNOS-pS1177 expression when exposed to UF. Upon enzymatic degradation of HS, exposure to UF results in no significant increase in eNOS-pS1177 expression, compared with the effects of static flow and DF. **q** Quantification of colocalization shows significant differences in eNOS-pS1177 overlap with cav-1 when RFPECs in UF conditions are compared to RFPECs in DF and static flow conditions. RFPECs exposed to static and DF conditions along with enzymatically degraded HS show lower cav-1 and eNOS-pS1177 colocalization that is statistically different to RFPECs with intact HS in UF conditions. **p**, **q** Number of independent experiments “N” are shown for each condition, and *P < 0.05, **P < 0.01, ***P < 0.001, and ****P < 0.0001 are indicated

We first investigated flow-dependent expression of eNOS-pS1177. Using fluorescence microscopy imaging and analysis, we found that static RFPEC monolayers had an average eNOS-pS1177 MFI of 39.3 ± 5.72 RFUs, which was normalized to 1 (Fig. 2b, p). Compared to static conditions, UF application resulted in a statistically significant 60.5 ± 12.1% increase in average eNOS-pS1177 MFI (Fig. 2h, p), to 55.8 ± 6.93 RFUs. DF application, compared to static conditions, resulted in a statistically insignificant 11.5 ± 13.4% decrease in eNOS-pS1177 MFI (Fig. 2e, p), to 32.4 ± 5.51 RFUs. Additionally, these results indicate a statistically significant near 50% lesser eNOS-pS1177 expression in cells exposed to DF compared to cells exposed to UF (Fig. 2e, h, p). These results were validated in vivo. Total eNOS was found to be substantially more expressed in the abdominal aorta (Fig. 4j), where blood flow is uniform, than the inner curvature of the distal aortic arch (Fig. 4i), where blood flow is disturbed. When quantified we determined that total eNOS exhibited a more than 25-fold increase in the abdominal aorta, as compared to the aortic arch.

We then analyzed cav-1 and eNOS-pS1177 colocalization in RFPECs and found that eNOS-pS1177 overlap with cav-1 was significantly elevated in UF conditions compared to static and DF conditions, as revealed by the Mander’s Overlap Coefficient analysis. Compared to static conditions, UF increased cav-1 colocalization with eNOS-pS1177 by 11.4 ± 3.25% (Fig. 2c, i, q). In contrast, exposure to DF decreased overlap by 1.31 ± 4.57% (Fig. 2c, f, q) when compared to static controls; however, this difference was not statistically significant. Comparing UF with DF, UF-treated cells had 12.9 ± 3.25% more cav-1 colocalization with eNOS-pS1177 (Fig. 2f, i, q). In other words, relevant to atheroprone conditions, these data indicate that DF statistically significantly impairs eNOS-pS1177 and cav-1 colocalization.

### The glycocalyx mechanosensor in vitro and in vivo: implications for flow-regulated caveolin-1 expression, distribution, and colocalization with eNOS

We hypothesized that the differential regulation of cav-1 expression, distribution, and colocalization with eNOS in areas of UF compared to DF were due to changes in the endothelial GCX, a known mechanosensor.

**Fig. 3:**
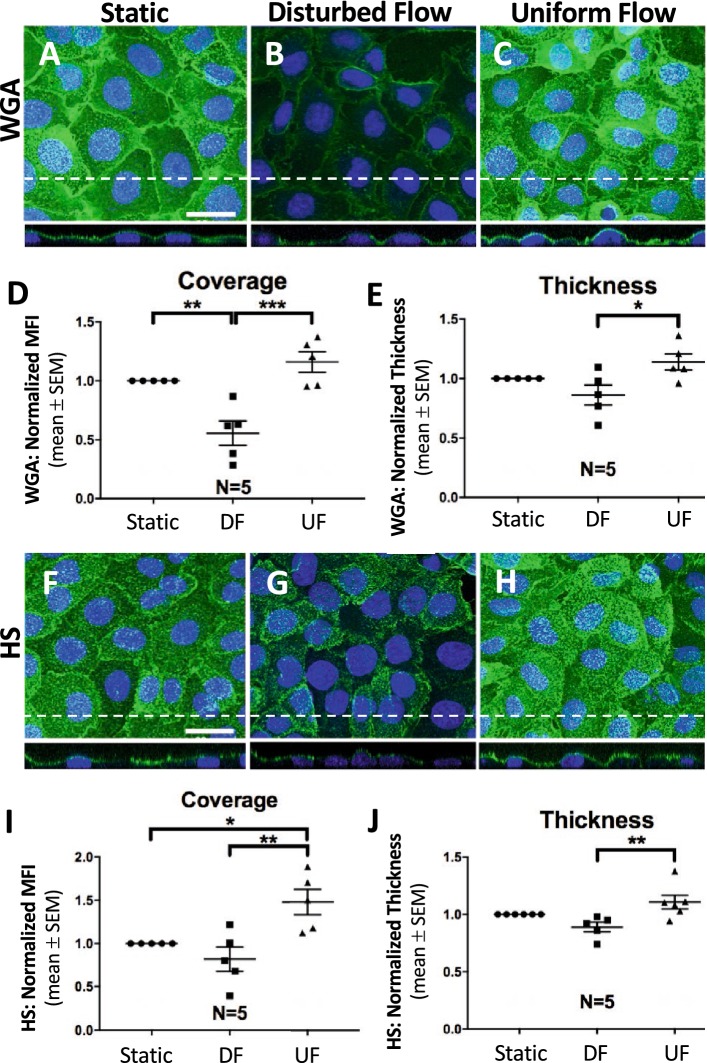
Disturbed flow decreases GCX expression and thickness. En face Z-projections and orthogonal views are shown for cells stained for WGA after exposure to **a** static, **b** DF, and **c** UF conditions. Cells were also stained with an antibody to tag HS in **f** static, **g** DF, and **h** UF conditions. **a**–**h** Dotted lines indicate the position of orthogonal views, and the scale bar = 30 μm. **d**, **e** Normalized WGA MFI (**d**), as an indicator of RFPEC coverage, and WGA thickness (**e**), determined as shown in Additional file 3: Fig. S3. **i**, **j** Similarly shows normalized HS (**i**) MFI/coverage and (**j**) thickness (also determined as shown in Additional file 3: Fig. S3). **d**–**j** Number of independent experiments “N” are shown for each condition, and *P < 0.05, **P < 0.01, and ***P < 0.001 are indicated

RFPEC monolayers were stained for GCX using WGA, a lectin that labels major GCX components including sialic acid and the *N*-acetylglucosamine monosaccharides of HS and hyaluronic acid [38, 78]. Staining revealed statistically significant differences in GCX immunofluorescence expression and GCX thickness in static versus UF versus DF conditions. RFPECs cultured in static conditions had a WGA MFI of 79.4 ± 14.8 RFUs and WGA thickness of 2.88 ± 0.265 µm (Fig. 3a, d, e). Compared to static controls, cells exposed to DF exhibited a statistically significant 44.4 ± 10.3% lesser WGA MFI and a statistically insignificant 13.8 ± 8.39% lesser thickness (Fig. 3b, d, e). Compared to static controls, exposure to UF resulted in an increase in both WGA MFI and WGA thickness, albeit statistically insignificant, by 16.0 ± 8.67% and 14.0 ± 6.76% (Fig. 3c–e). When cells exposed to UF were compared to cells exposed to DF, WGA MFI and thickness in UF conditions were found to be statistically significantly higher by 108 ± 8.67% and 32.3 ± 6.76%, respectively (Fig. 3b–e).

When we stained RFPEC GCX using another approach, an antibody targeted only to the HS GCX component, we re-confirmed the trend by which flow induces changes in GCX. Static RFPECs had an HS MFI of 36.2 ± 10.5 RFUs and HS thickness of 2.54 ± 0.460 µm (Fig. 3f, i, j). Compared to static conditions, exposure to DF resulted in a decrease in HS MFI and HS thickness by 18.0 ± 14.1% and 10.9 ± 4.25%, respectively, although the decreases were statistically insignificant (Fig. 3g, i, j). Exposure to UF resulted in a statistically significant 48.0 ± 14.8% increase in HS MFI and a statistically insignificant 10.8 ± 5.95% increase in HS thickness, when compared to static control HS (Fig. 3h–j). Notably, cells exposed to UF, compared to DF, showed statistically significant 80.4 ± 14.8% elevated HS MFI and 24.4 ± 5.95% increased HS thickness (Fig. 3g–j).

**Fig. 4:**
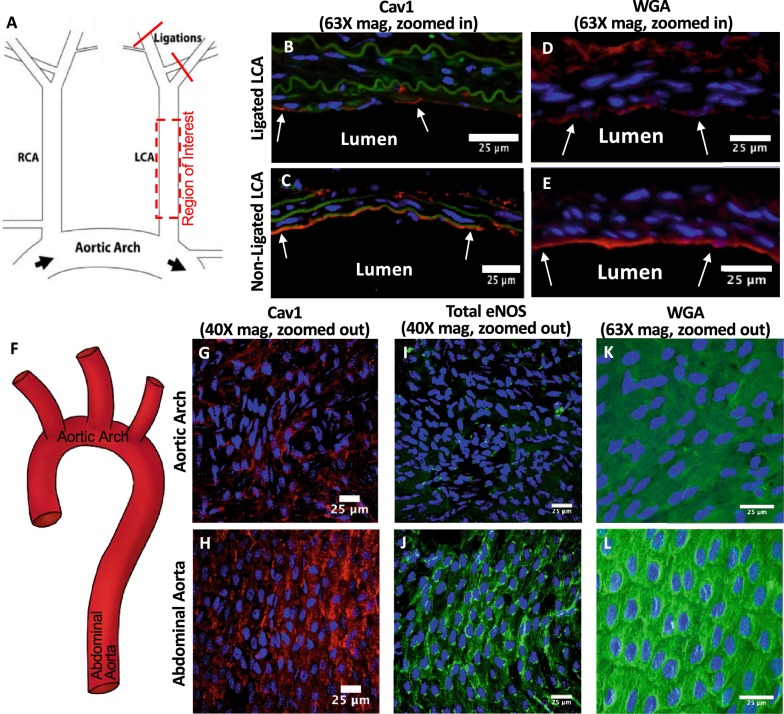
Flow-dependent regulation of cav-1, eNOS, and GCX is confirmed in vivo. **a** Schematic of the partial carotid ligation model in which three of the four caudal branches of the LCA are ligated, generating acute DF within the LCA. **b** DF induced by partial ligation decreases cav-1 (red) expression in the LCA, when compared to (**c**) non-ligated LCAs. Nuclei were stained using DAPI (blue) while autofluorescence from elastin was also imaged (green). **d** DF induced by partial ligation also decreases GCX expression as determined by WGA staining when compared to (**e**) non-ligated LCAs. **f** Schematic of the aorta, showing the inferior curvature of the aortic arch and the abdominal aorta where ECs were examined. **g**–**l** The expression of cav-1, eNOS, and WGA is decreased in the aortic arch (**g**, **i**, **k**) when compared to the abdominal aorta (**h**, **j**, **l**), respectively

The effects of UF and DF on GCX expression in vivo were similar to what we found in vitro. Compared to UF conditioned non-ligated LCAs (Fig. 4e), acute DF-conditioned ligated LCAs (Fig. 4d) exhibited weaker and spottier expression of the GCX as determined by WGA. Analysis of WGA also revealed that the inner curvature of the aortic arch (Fig. 4c) expressed a lesser amount of GCX than the UF-conditioned abdominal aorta (Fig. 4l). These results suggest that DF environments elicit weaker GCX expression than UF environments, which promote a highly expressed, continuous GCX layer. Combined with analysis of cav-1 and total eNOS expression on mouse LCAs and aortas exposed to different flow conditions, these results point to a known mechanosensory, GCX, as a responsible agent for differential expression of cav-1 and eNOS in relation to atherosclerosis development.

To test this hypothesis, we determined if there was a causal relationship between GCX expression and flow-regulated cav-1 expression, distribution, and colocalization with eNOS-pS1177. To do this, we investigated the effects of UF conditions on RFPECs treated with 62.5 µIU/mL of Hep III, which enzymatically degrades HS. The enzyme treatment decreased RFPEC HS MFI by approximately 50% in static conditions and approximately 60% in UF conditions (Additional file 2: Fig. S2). RFPEC HS thickness was enzymatically decreased by approximately 35% in static conditions and approximately 40% in UF conditions (Additional file 2: Fig. S2).

In static conditions, HS degradation did not affect cav-1 expression, eNOS-pS1177 expression, or eNOS-pS1177 overlap with cav-1. To be specific, compared to non-treated static samples, HS deficient static samples showed a 5.3% decrease in cav-1 expression (Fig. 1a, d, f), a 5.5% decrease in eNOS-pS1177 expression (Fig. 2b, k, p), and no change in cav-1 overlap by eNOS-pS1177 (Fig. 2c, l, q). These changes were statistically insignificant. In addition, preferential localization of cav-1 to the cell borders of static samples was retained with Hep III treatment. Evidence of this came from comparing Hep III-treated static samples to non-treated static samples and finding that kurtosis analysis demonstrated a statistically insignificant 7.6 ± 3.1% increase in cav-1 centralization to cell borders (Fig. 1i, j). More noticeable were the changes in cav-1 distribution related to flow direction after Hep III incubation. While in non-treated static conditions cav-1 was diffusely distributed in 43.7 ± 4.31% of cells, only 20.0 ± 3.57% of Hep III treated static cells had diffusely distributed cav-1. This difference was found to be statistically significant (Fig. 1j). In short, cell treatment with Hep III induces cav-1 preference for distribution to no particular cellular zone.

The effects of HS degradation were more apparent in UF conditions, implicating the role of HS in force-dependent regulation of EC function. Degradation of HS significantly blocked UF-induced increase in cav-1 expression, cav-1 preferential distribution to the upstream cellular zone, and eNOS-pS1177 overlap of cav-1.

Non-treated UF cells expressed increased cav-1, by over 60%, compared to non-treated static samples. However, in Hep III-treated cells, UF resulted in a 1.0 ± 17.6% decrease in cav-1 expression compared to non-treated static samples (Fig. 1f). Post-hoc analysis demonstrated that the difference in cav-1 expression between non-treated and Hep III-treated cells exposed to UF was statistically significant (Fig. 1f).

HS degradation also inhibited UF-induced changes in cav-1 cellular distribution (Fig. 1g–j). In contrast to non-Hep III treated samples, cells exposed to UF after enzymatic HS degradation did not experience a significant increase in cav-1 preference for upstream cellular localization when both are compared to untreated static conditions (Fig. 1i, j). Hep III-treated cells exposed to UF had a 0.9 ± 3.3% decrease in upstream cav-1 localization compared to untreated static samples. This corresponds to a statistically significant 54.3 ± 3.3% decrease in upstream localization, between non-treated and Hep III-treated cells exposed to UF (Fig. 1j). HS degradation did not impact cav-1 centralization to cell borders in UF conditions, when compared to conditions of intact HS under UF. Specifically, cells with an intact HS layer experienced a 6.2 ± 2.6% UF-induced increase in kurtosis value, compared to untreated static conditioned cells (Fig. 1g, h), while HS deficient samples showed decreased kurtosis, by 2.5 ± 3.7%, compared to untreated static samples (Fig. 1g, h).

Finally, HS degradation impaired UF-induced increases in eNOS-pS1177 and its colocalization with cav-1 (Fig. 2h, i, n–q). In untreated samples, UF increased eNOS-pS1177 expression by 60.5 ± 12.1% and cav-1 colocalization with eNOS-pS1177 by 11.4 ± 3.25% compared to static controls. In contrast, Hep III treated samples were statistically similar to untreated static samples, with Hep III treated static samples showing only 0.01% less eNOS-pS1177 colocalization with cav-1 and Hep III treated UF samples showing a 0% change in eNOS-pS1177 colocalization with cav-1 (Fig. 2c, l, o, q). When compared to untreated UF samples, eNOS-pS1177 colocalization with cav-1 was decreased by 10.2% in Hep III treated UF samples (Fig. 2i, o, q).

## Discussion

Our goal was to link well-defined physiologically accurate UF and DF conditions to EC phenotype that is atherosclerosis-resistant or atherosclerosis-permissive, respectively, as mediated by the GCX mechanotransducer. In the end, we confirmed that in the presence of DF patterns and shear stress levels that are characteristic of atheroprone areas of the vasculature, GCX expression is weak and is a key factor underlying inhibition of EC cav-1 expression and eNOS activity, which were taken as indicators of pro-atherosclerotic EC phenotype.

Specifically, we found that in vitro exposure of ECs to anti-atherosclerotic UF led to increased expression of the principal caveolae marker, cav-1, preference for cav-1 localization to the upstream zone of the cellular body, and cav-1 distribution to EC-to-EC appositions. The response of cav-1 to atheroprotective UF, compared to static conditions, is somewhat established. Rizzo et al. [21] previously found that exposure of bovine aortic ECs to UF increased cav-1 by fivefold, compared to static controls. Sun et al. [20] showed a significant increase in cav-1 localization at the upstream side of cell bodies in UF compared to static conditions. Our observed significant UF-induced increases in cav-1 expression (Fig. 1a, c, f) and in cav-1 localization at the upstream side of cell bodies (Fig. 1j) agree with the previous findings [20, 21]. To our knowledge, while decreased expression of cav-1 has been observed in human atherosclerotic plaques and is associated with atheroma formation [86–88], there has been no direct evidence of how cav-1 is affected by DF, which is viewed as an initiator of endothelial dysfunction in the onset of atherosclerosis. Here, we found that exposure of RFPECs to DF, compared to UF, results in decreased cav-1 expression (Fig. 1b, c, f). Furthermore, using our novel atherosclerosis-relevant parallel plate flow chamber, we observed de-localization of cav-1 from RFPEC borders and the upstream side of cell bodies compared to UF (Fig. 1g–f). These findings were corroborated by in vivo data that demonstrated decreased cav-1 expression in ligated LCAs affected by acute DF (Fig. 4b) and the inner curvature of the distal aortic arch affected by chronic DF (Fig. 4g) when compared to UF conditioned controls that include the non-ligated LCA and proximal abdominal aorta, respectively (Fig. 4c, h). Taken together with previous studies indicating a vasculoprotective role of cav-1 and caveolae [22, 82–84], our in vitro and in vivo findings suggest that atheroprone flow can lead to endothelial dysfunction through decreased cav-1/caveolae expression and localization.

To determine the functional consequences of differential UF versus DF regulation of cav-1, we considered that endothelial caveolae have been implicated in a variety of cellular signaling processes including NO signaling [22, 82–84, 89, 90]. Therefore, we investigated the effects of DF and UF on eNOS-pS1177, which is an active form of eNOS that resides in caveolae and synthesizes and releases NO to promote endothelial health. In agreement with previous findings, we found eNOS-pS1177 levels and eNOS-pS1177 colocalization with cav-1 to be significantly elevated in regions of UF [21]. Our studies confirmed our expectation that in DF conditions, where cav-1 expression is disrupted, eNOS-pS1177 expression and colocalization with cav-1 are reduced. In comparison to UF, eNOS-pS1177 expression in regions of DF decreased near twofold. Our observation of eNOS-pS1177 expression in RFPECs and total eNOS in mouse vessels agree with previous studies [35, 74, 91, 92]. For example, Won et al. [35] found that the application of 72 h of UF significantly increased eNOS-pS1177 expression compared to DF. While the study performed by Won et al. exposed cells to an extended time period compared to our study, it has been shown that flow-induced changes in NO can occur within several minutes of flow application [53, 93]. We exposed RFPECs to a 6-h period and found that changes in eNOS-pS1177 expression occur within the 6-h time frame. We also observed a similar upward trend in expression of total eNOS in UF-conditioned mouse abdominal aortas compared to DF conditioned mouse aortic arches (Fig. 4i, j). More specifically, we found a 28-fold decrease in eNOS expression in areas of DF (aortic arch/descending aorta) than in regions of UF (proximal abdominal aorta).

Rizzo et al. [21] previously found that the application of UF resulted in increased residence of eNOS-pS1177 within caveolar compartments of ECs. However, this study did not probe caveolae in regions of DF. Here, we expanded on the findings of Rizzo et al., demonstrating that colocalization of cav-1 with eNOS-pS1177 is significantly higher in cells exposed to UF when compared to regions of DF (Fig. 2). Due to its role in eNOS activation, the observed increase in cav-1 overlap with eNOS-pS1177 suggests that decreases in eNOS-pS1177 in DF are at least partially a result of the flow regulation of cav-1-eNOS colocalization. Decreased expression of cav-1 in areas of DF may also further exacerbate this phenomenon. In regards to these findings, it is important to note that cav-1 has been shown to inhibit eNOS activity [28–31]. Despite this, in the present study we do not expect direct, molecular interaction between the two molecules in vitro because such studies investigated the active, phosphorylated form of eNOS, which does not bind to cav-1 [94]. Instead, colocalization of eNOS-pS1177 with cav-1 is an indicator of its presence within caveolae, which agrees with previous studies. However, we cannot speculate on the in vivo interaction between cav-1 and eNOS as we did not perform *en face* co-staining and used an antibody targeting total eNOS. Additionally, while this study only addressed the impact of cav-1 deregulation on NO production, it is possible that DF-induced cav-1 deregulation impacts additional cav-1 signaling pathways. Nevertheless, the collective data from this study indicates that the loss of caveolae/eNOS-pS1177 colocalization and subsequent decreased eNOS activation in areas of DF may further increase the risks for atherosclerosis development.

In this study, immunoprecipitation and proximity ligation assays were not performed due to our interest in identifying eNOS localization within caveolae without direct interaction between eNOS and cav-1, which is believed to inhibit eNOS activity [28–31]. Isolation of endothelial plasma membranes and subsequent isolation of the caveolae fraction of the plasma membrane to then detect eNOS within the caveolae, a technique established by Rizzo et al. [95], were also not performed. Localization of eNOS within caveolae was evaluated using immunofluorescence in place of these other methods because previous immunofluorescence findings were corroborated using transmission electron microscopy (TEM) and western blotting of subfractionated luminal plasma membrane [21]. In addition, other similar correlative studies using immunofluorescence, Western blotting, immunoprecipitation, and TEM validated colocalization of caveolae with additional cellular components including dynamin [96] and the transforming growth factor-β (TGF-β) type I receptor [also known as activin receptor-like kinase (ALK)] [97]. These studies, all taken together, suggest that localization of eNOS in caveolae can be accurately determined using the immunofluorescence technique.

In addition to studying cav-1 expression and colocalization with eNOS, as an indicator of EC phenotype, we sought to also identify an upstream mechanism of flow-mediated cav-1 expression and function. Previous research has shown that the endothelial GCX is weakly expressed in areas of atherosclerosis [5–7], implicating the endothelial GCX in pro-atherosclerotic EC dysfunction, for example, by deregulating vasculoprotective molecules such as NO [51, 53, 55]. Previously, Koo et al. [49] used a cone-and-plate flow apparatus to study GCX regulation after exposure to 24 h of UF or DF. They found that GCX expression is increased in regions of UF but decreased in areas of DF. However, their study isolated cells in DF from those in UF, preventing communication between the two cell phenotypes as is experienced in vivo. This current study uses a more physiologically relevant parallel-plate flow chamber with juxtaposed regions of DF and UF, allowing for proper EC communication. From our RFPEC studies we also demonstrated that changes in GCX expression can occur in as little as 6 h of flow exposure, suggesting that the GCX may help regulate rapidly changing endothelial processes. In the present study, we specifically investigated the effects of pro-atherosclerotic flow on the sialic acid, hyaluronic acid, and HS components of the GCX, labeled using WGA lectin and HS antibody. The specific GCX components of interest were chosen due to previous research implicating them in NO regulation [52, 54, 56, 98]. We found that RFPEC exposure to DF resulted in statistically significant decreases in GCX coverage and thickness (Fig. 3). Similarly, mouse LCAs and aortas demonstrated similar decreases in GCX expression in the presence of DF (Fig. 4). Furthermore, we observed that the extent of GCX decrease (as well as cav-1 and eNOS differences) was spatially variable when we compared the proximal versus distal aortic arch (data not presented), indicating the DF vascular regions are areas of transitional endothelial phenotype.

By applying Hep III enzyme to HS, a major component of the GCX, we were able to ascertain whether decreased GCX expression in DF regions has a causal relationship with cav-1 and eNOS-pS1177. Interestingly, we found that HS degradation eradicated the effects of UF on cav-1 expression and distribution (Fig. 1). Under UF conditions with an intact GCX, cav-1 expression was significantly increased as was upstream and appositional localization. In contrast, no changes in expression or upstream localization were observed in Hep III-treated UF-conditioned cells when compared to Hep III controls, while appositional localization was retained. The findings of unchanged appositional localization of cav-1 in the absence of HS suggest that the localization of caveolae to cell–cell borders is not dependent on HS. However, all of our other evidence points to the fact that the extent and upstream nature of cav-1 expression are dependent on the presence of the HS component of the GCX. While previous research has suggested that caveolae serve as primary mechanosensors to shear stress and assist in eNOS activation [99, 100], the work presented here indicates that caveolae act as indirect mechanosensors that are mediated by HS. Therefore, we propose that decreased cav-1 and caveolae polarization in ECs associated with atherosclerotic plaques is due to poor expression of HS. Additionally, other GCX components, such as hyaluronic acid, may similarly regulate cav-1 expression and localization, as the hyaluronic acid receptor CD44 is localized to caveolae [101]. However, this was outside the scope of the current study.

We also found that HS is necessary for UF-mediated eNOS activation, showing significantly less eNOS-pS1177 expression in Hep III-treated UF-conditioned cells than UF-conditioned cells with an intact GCX (Fig. 2). These findings agree with one of our previous reports, which shows HS as a necessary GCX component for eNOS activation [54] and proposes a GCX-caveolae-eNOS pathway in which under UF, the HS glycosaminoglycans, bound to the glypican core protein GCX, activate eNOS through their association with caveolae [54, 102]. More interestingly, we found, for the first time, that HS degradation resulted in decreased cav-1 and eNOS-pS1177 colocalization (Fig. 2). These results suggest that decreased eNOS activation in DF regions is a result of inadequate caveolae-eNOS colocalization due to decreased HS expression. Furthermore, our data imply that HS can trigger post-translational modifications of eNOS or cav-1, such as eNOS acetylation, which is implicated in cav-1-eNOS colocalization [31]; although, confirming acetylation is beyond the scope of the current study.

## Conclusion

**Fig. 5:**
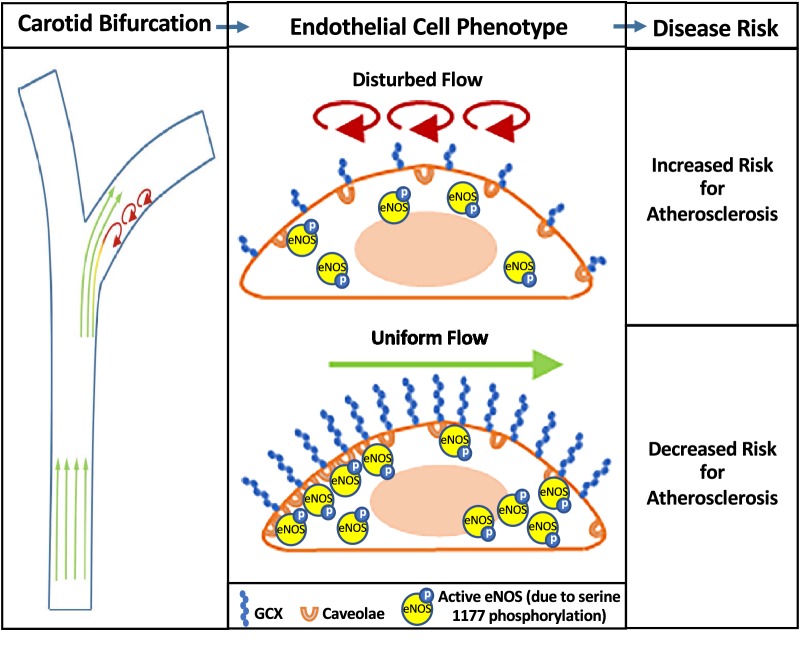
Conceptual model: In the carotid bifurcation, a common area for atherosclerosis development, disturbed flow causes GCX-mediated impairment of caveolae expression and eNOS-associated function. Specifically, in DF conditions GCX is diminished and, in turn, causes caveolae to be scarce and to exhibit diffuse localization, reduces eNOS-pS1177 expression, and disrupts caveolae and eNOS-pS1177 colocalization. These events are likely to weaken NO signaling and increase the risk of endothelial-dependent atherosclerosis. In UF conditions GCX is healthy and intact. As a result, caveolae is abundant and exhibits localization that is polarized to the subcellular region upstream of the flow direction. This expression and distribution of the caveolae coincides with elevated eNOS-pS1177 expression and colocalization with eNOS-pS1177, which is necessary for anti-atherosclerotic NO signaling

In summary, our study demonstrates that DF can lead to EC dysfunction associated with atherosclerosis by inhibiting GCX expression and regulation of cav-1 (Fig. 5). Additionally, exposure to DF blocks eNOS activation and association with caveolae due to loss of HS, a major GCX component (Fig. 5). Together, these results suggest that GCX deterioration promotes a pathological EC phenotype and vascular dysfunction that can lead to atherosclerosis (Fig. 5). Considering that atherosclerosis accounts for up to 1 in every 3 deaths in the United States [1], future development of new cardiovascular therapies aimed to prevent GCX deterioration or support GCX regeneration will be worthwhile.

## Additional files

**Additional file 1: Fig. S1.** Custom parallel-plate flow chamber creates physiologically relevant disturbed flow patterns. (A) Typical flow patterns in the carotid artery and carotid bifurcation. DF patterns within the carotid sinus are characterized by recirculation. (B) Expanded view of the flow apparatus, including the rubber gasket (red) used to create the flow channel. The black box indicates the step referenced in Fig. 1d. (C) Shear stress values experienced by cells along the bottom plate, with the x-axis being the distance from the downstream edge of the step to the cells in question. (D) Orthogonal view of the velocity profile throughout the chamber. (E) Heat map correlating to flow velocities (cm/s) pictured in Fig. 1d.

**Additional file 2: Fig. S2.** Heparinase III degrades heparan sulfate thickness and coverage by ~50%. *En face* Z-projections of HS stained RFPECs (A) in static control, (B) exposed to Hep III in static conditions, (C) exposed to Hep III under UF, (D) exposed to DF, and (E) exposed to UF. (F) Quantification of normalized HS coverage as determined by MFI. (G) Quantification of normalized HS thickness. Indicating statistical significance, * = p<0.05 and ** = p<0.01.

**Additional file 3: Fig S3.** Demonstration of GCX thickness analysis. Orthogonal views were obtained from z-stack images. Intensity profiles across the GCX layer were then created using the line tool and the width of the intensity profile was measured.

**Additional file 4: Fig. S4.** Caveolin-1 is preferentially located at the upstream side of cells exposed to uniform flow. (A) Quantification of cav1 distribution in relation to flow direction for cells in (A) static flow, (B) DF, or (C) UF conditions. (D) Comparison of cav1 distribution between various zones of flow-conditioned cells: upstream, downstream, upper lateral, and lower lateral. Cells that have no preferential cav-1 distribution are considered to have diffuse distribution. *P<0.05, **P<0.01, ***P<0.001, and ****P<0.0001.

## Abbreviations

BSA: bovine serum albumin
Cav-1: caveolin-1
Cy3: cyanine-3
DAPI: 4′6-diamidino-2-phenylindole
DF: disturbed flow
DMEM: Dulbecco’s Modified Eagle Medium
EC: endothelial cell
eNOS: endothelial-type nitric oxide synthase
eNOS-pS1177: serine 1177 phosphorylated endothelial-type nitric oxide synthase
FBS: fetal bovine serum
GCX: glycocalyx
H_2_O_2_: hydrogen peroxide
Hep III: heparinase III
HS: heparan sulfate
HRP: horseradish peroxidase
LCA: left carotid artery
MFI: mean fluorescence intensity
NO: nitric oxide
OCT: optimum cutting temperature
PBS: phosphate buffered saline
P/S: penicillin streptomycin
RFPECs: rat fat pad endothelial cells
SEM: standard error of the mean
UF: uniform flow
WGA: wheat germ agglutinin
