# Supplementary figures and images for: Pro-atherosclerotic disturbed flow disrupts caveolin-1 expression, localization, and function via glycocalyx degradation
Source: J Transl Med. 2018 Dec 18;16:364. doi: 10.1186/s12967-018-1721-2 (PMC6299559; doi:10.1186/s12967-018-1721-2)

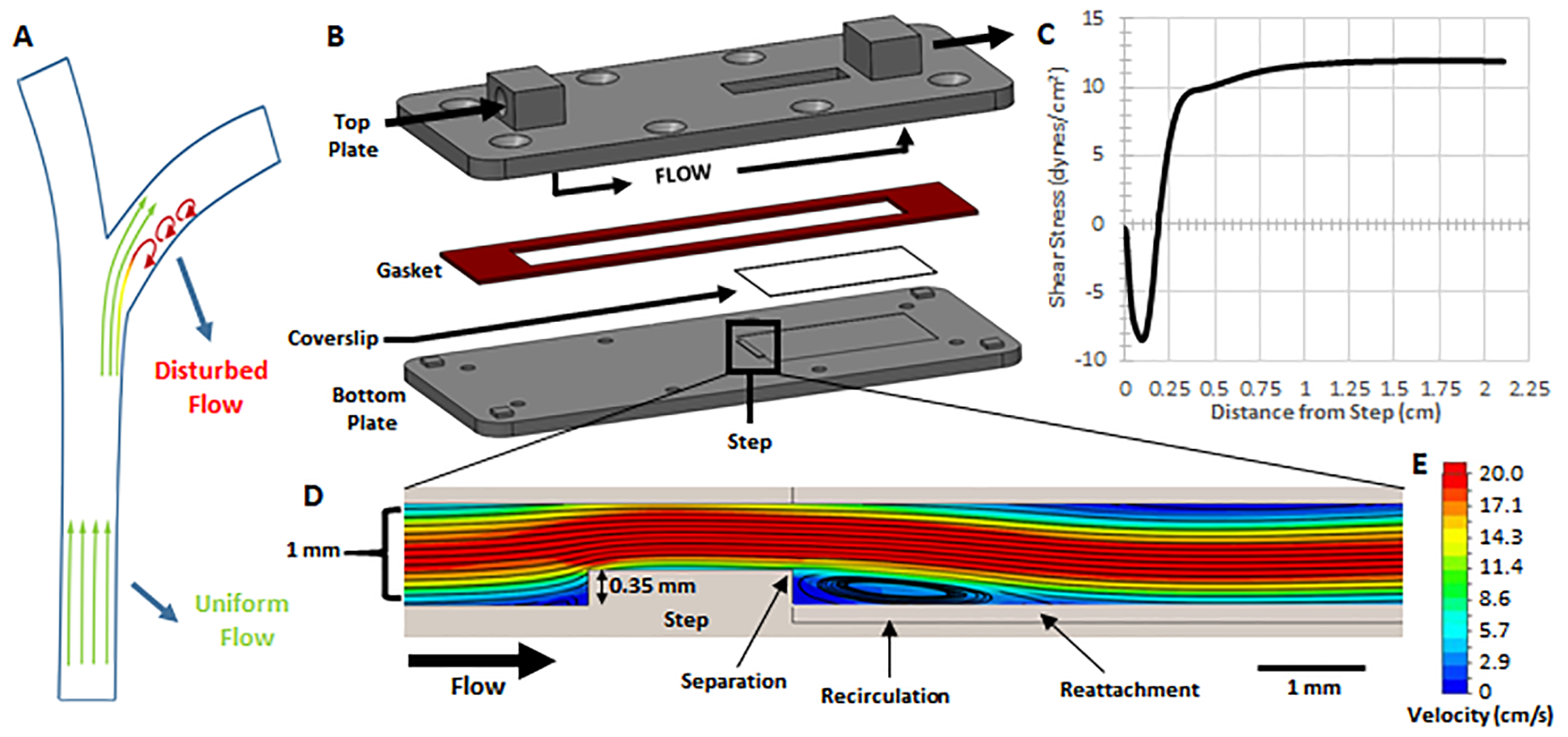

Supplement: Supplementary file 1 — Additional file 1: Fig. S1. Custom parallel-plate flow chamber creates physiologically relevant disturbed flow patterns. (A) Typical flow patterns in the carotid artery and carotid bifurcation. DF patterns within the carotid sinus are characterized by recirculation. (B) Expanded view of the flow apparatus, including the rubber gasket (red) used to create the flow channel. The black box indicates the step referenced in Fig. 1d. (C) Shear stress values experienced by cells along the bottom plate, with the x-axis being the distance from the downstream edge of the step to the cells in question. (D) Orthogonal view of the velocity profile throughout the chamber. (E) Heat map correlating to flow velocities (cm/s) pictured in Fig. 1d. [file 12967_2018_1721_MOESM1_ESM.tif]

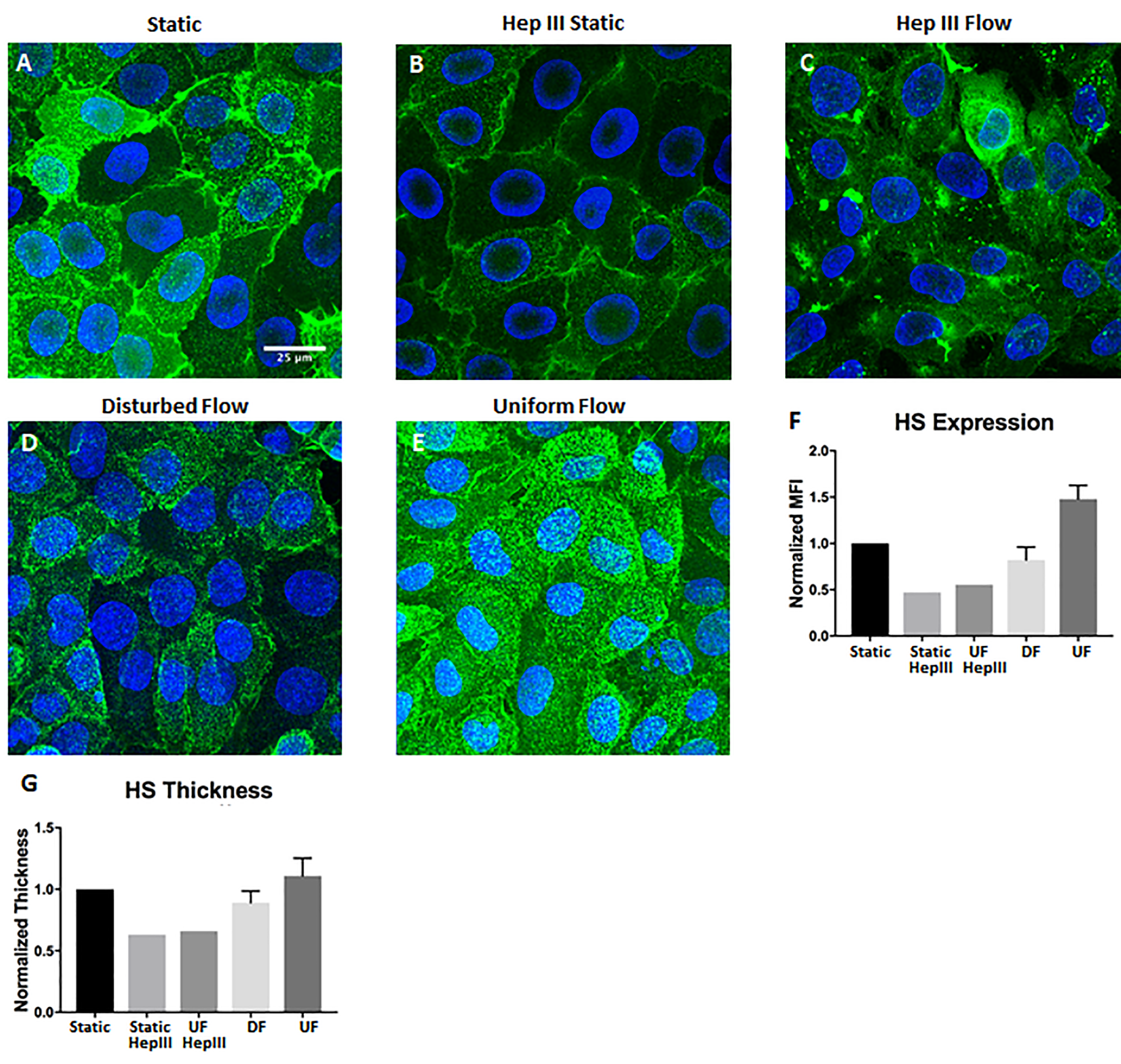

Supplement: Supplementary file 2 — Additional file 2: Fig. S2. Heparinase III degrades heparan sulfate thickness and coverage by ~50%. En face Z-projections of HS stained RFPECs (A) in static control, (B) exposed to Hep III in static conditions, (C) exposed to Hep III under UF, (D) exposed to DF, and (E) exposed to UF. (F) Quantification of normalized HS coverage as determined by MFI. (G) Quantification of normalized HS thickness. Indicating statistical significance, * = p<0.05 and ** = p<0.01. [file 12967_2018_1721_MOESM2_ESM.tif]

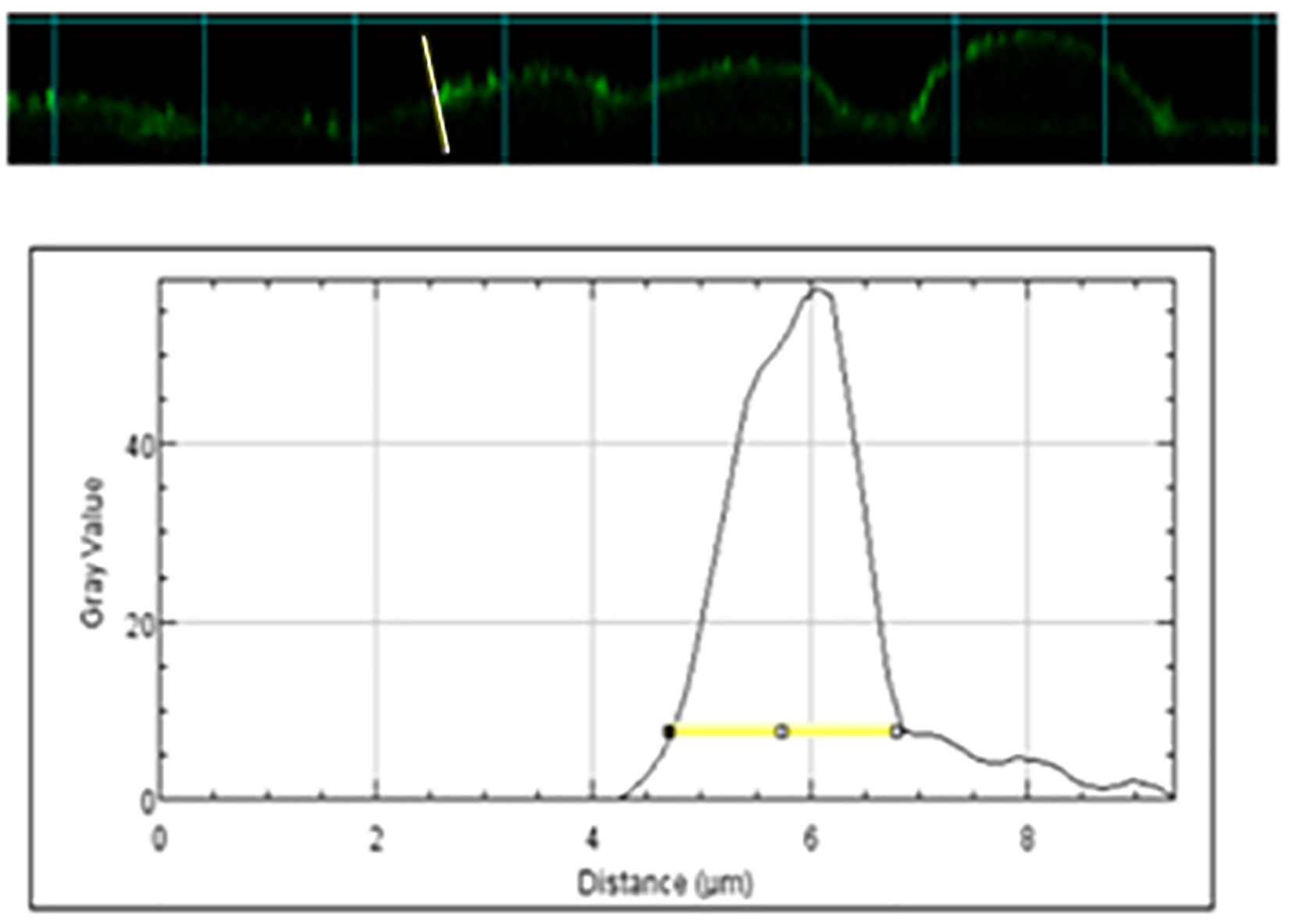

Supplement: Supplementary file 3 — Additional file 3: Fig S3. Demonstration of GCX thickness analysis. Orthogonal views were obtained from z-stack images. Intensity profiles across the GCX layer were then created using the line tool and the width of the intensity profile was measured. [file 12967_2018_1721_MOESM3_ESM.tif]

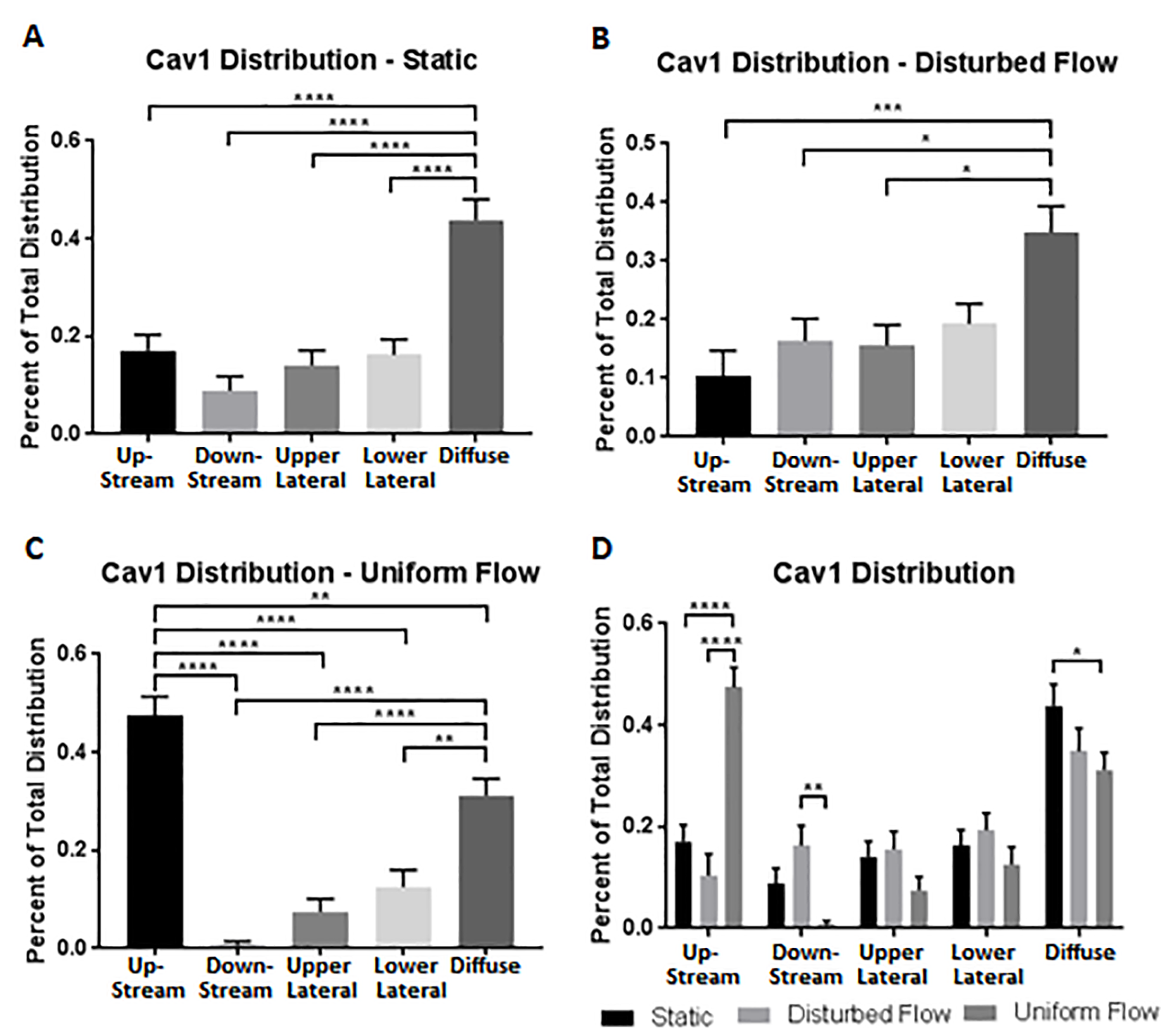

Supplement: Supplementary file 4 — Additional file 4: Fig. S4. Caveolin-1 is preferentially located at the upstream side of cells exposed to uniform flow. (A) Quantification of cav1 distribution in relation to flow direction for cells in (A) static flow, (B) DF, or (C) UF conditions. (D) Comparison of cav1 distribution between various zones of flow-conditioned cells: upstream, downstream, upper lateral, and lower lateral. Cells that have no preferential cav-1 distribution are considered to have diffuse distribution. *P<0.05, **P<0.01, ***P<0.001, and ****P<0.0001. [file 12967_2018_1721_MOESM4_ESM.tif]
